# Lycopene attenuates dexamethasone induced depression like behavior and immunological dysfunction via restoration of neuro-immune-metabolic homeostasis in rats

**DOI:** 10.1038/s41598-026-62143-9

**Published:** 2026-07-19

**Authors:** Hend A. Essa

**Affiliations:** Nutrition and Food Sciences Department, Food Industries and Nutrition Research Institute, National Research Centre, 33 El Bohouth St, Dokki, Giza, 12622 Egypt

**Keywords:** Dexamethasone, Lycopene, Depression-like behaviors, Immunosuppression, Carotenoid, Oxidative stress, Neuroinflammation, Diseases, Immunology, Neurology, Neuroscience

## Abstract

Chronic glucocorticoid therapy, while clinically indispensable, often induces debilitating neuropsychiatric side effects, including depression-like behaviors, alongside systemic immunosuppression and metabolic dysfunction. The convergence of central neurotoxicity and peripheral immune dysregulation presents a unique therapeutic challenge requiring multi-target interventions. This study investigated the prophylactic efficacy of lycopene against dexamethasone-induced behavioral, neurochemical, oxidative, inflammatory, and immunological perturbations in rats. Thirty-two adult male Sprague-Dawley rats were randomly assigned to four groups (*n* = 8): normal control, dexamethasone (2 mg/kg/day/intraperitoneal), lycopene (10 mg/kg/day/orally), and dexamethasone+lycopene combination. Treatments were administered daily for 21 days. Behavioral assessments (forced swim test, tail suspension test) were conducted. In frontal cortex and hippocampal tissues, neurochemical markers (brain-derived neurotrophic factor, gamma-aminobutyric acid, and acetylcholinesterase activity), neurotransmitters (serotonin, dopamine), oxidative stress markers (malondialdehyde, nitric oxide, reduced glutathione, superoxide dismutase, catalase), inflammatory cytokines (tumor necrosis factor-alpha and interleukin-6), and caspase-3 were quantified. In serum, fasting blood glucose, insulin, liver and kidney function markers, oxidative stress markers, inflammatory cytokines (interleukin-4, tumor necrosis factor-alpha, interferon-gamma), cluster of differentiation 4 and 8 concentrations (CD4, CD8), and complete blood count were analyzed. Dexamethasone significantly (*p* < 0.05) increased immobility time in behavioral tests, depleted serotonin and dopamine, elevated oxidative stress markers, and induced leukopenia with disrupted CD4/CD8 ratios. Lycopene co-treatment reversed behavioral despair, restored neurotransmitter homeostasis, attenuated oxidative-inflammatory cascades, normalized immune cell populations, and improved metabolic parameters. Lycopene provides integrated multi-system protection against glucocorticoid-induced neurobehavioral and immunological dysfunction, positioning it as a potential promising nutritional adjunct for patients requiring chronic corticosteroid therapy.

## Introduction

Depression is defined as a widespread chronic medical illness that can affect physical and mental health^1^. According to the World Health Organization (WHO), approximately 350 million people worldwide suffer from depression, making it a leading cause of disability^2^.

Depression is a debilitating global health disorder whose complex etiology is now recognized to extend far beyond monoaminergic deficits. Contemporary pathophysiological models emphasize a triad of chronic neuroinflammation, heightened oxidative stress, and dysregulation of the hypothalamic-pituitary-adrenal (HPA) axis. Hyperactivity of the HPA axis leads to sustained glucocorticoid elevation, which, in a paradox of physiology, becomes neurotoxic, inducing oxidative damage, suppressing neurotrophic support, and activating microglia-driven pro-inflammatory cascades in mood-regulating regions like the hippocampus and prefrontal cortex^3,4^. This destructive interplay creates a vicious cycle that compromises neuronal integrity and synaptic plasticity, forming the biological substrate of depressive symptomatology.

Synthetic glucocorticoids, particularly dexamethasone (Dex), are indispensable in clinical practice for their potent anti-inflammatory and immunosuppressive properties^5^. However, prolonged administration presents a paradox: while therapeutically valuable, Dex frequently induces neuropsychiatric side effects including depression-like behaviors, cognitive deficits, and immunosuppression^6^. Experimentally, chronic Dex exposure in rodents provides a robust translational model that recapitulates this dual pathology, reliably inducing oxidative stress, neurochemical disturbances, and immune dysfunction^7^. This model underscores an urgent need for adjunctive therapies capable of mitigating glucocorticoid-induced neurotoxicity while preserving immune competence.

In this context, in the search for safe, multi-target interventions, lycopene, a naturally occurring carotenoid abundant in tomatoes and red fruits, has emerged as a promising candidate. Its potent bioactivity is derived from its unique lipophilic structure, conferring superior capacity to quench singlet oxygen and neutralize reactive oxygen and nitrogen species^8^. Beyond direct antioxidant effects, lycopene exerts significant anti-inflammatory actions, including the downregulation of pivotal pro-inflammatory cytokines such as TNF-α and IL-6, and inhibition of the NF-κB signaling pathway^9^. Critically, lycopene crosses the blood–brain barrier, with documented brain bioavailability that supports its direct neuroprotective actions^10^. A growing body of preclinical evidence demonstrates its efficacy in mitigating depression-like phenotypes in various stress models, linked to mechanisms such as the upregulation of hippocampal brain-derived neurotrophic factor (BDNF), attenuation of apoptosis, and modulation of monoaminergic and GABAergic systems^11,12^.

Despite accumulating evidence supporting lycopene’s neuroprotective and anti-inflammatory properties, its capacity to counteract the neurobehavioral and immunological consequences of chronic glucocorticoid exposure remains largely unexplored. Existing studies have predominantly focused on metabolic dysfunction, generalized oxidative stress, or non-pharmacological stress paradigms, leaving a critical gap in understanding whether lycopene can mitigate the clinically relevant, iatrogenic toxicity induced by long-term dexamethasone administration. Importantly, glucocorticoid-induced depression represents a unique pathological entity characterized by the convergence of central neurotoxicity and systemic immunosuppression a dual burden that has rarely been addressed within a unified experimental framework.

Accordingly, the present study was designed to test the hypothesis that prophylactic lycopene supplementation provides integrated protection across the neuro–immune–metabolic axis during chronic dexamethasone exposure. The principal novelty of this work lies in its comprehensive, multi-level evaluation of behavioral outcomes, region-specific neurochemical and neurotrophic alterations, oxidative and inflammatory brain injury, systemic immune suppression, and metabolic organ dysfunction within a single, translationally relevant model. By employing validated behavioral paradigms (forced swim test, tail suspension test) alongside markers of oxidative stress (MDA, SOD, GSH, catalase, NO), neuroinflammation (IL-6, TNF-α, caspase-3), neurotransmission (serotonin, dopamine, GABA, acetylcholinesterase), systemic immunity (WBC subsets, CD4/CD8 ratio, cytokine profiles), as well as hepatic, renal, and metabolic function parameters, this work aims to provide comprehensive mechanistic insights into lycopene’s neuroprotective, immunomodulatory, and metabolic protective efficacy against dexamethasone-induced toxicity.

## Materials and methods

### Materials

#### Chemicals and natural products

Dexamethasone utilized in this investigation was obtained from EIPICO (10th of Ramadan City, Egypt) and subsequently diluted with normal saline prior to administration. Lycopene was acquired from Sigma-Aldrich Chemical Company. Fresh lycopene solutions were prepared immediately before use by dissolving in corn oil, which served as the vehicle^13^.

#### Animals

Adult male Sprague Dawley rats, aged between 6 and 8 weeks with body weights ranging from 200 to 240 g, were sourced from the Animal Health Research Institute, Egypt. Upon arrival, the animals were given a two-week acclimatization period within the institutional animal facility under carefully regulated environmental conditions. Throughout the experimental timeline, rats were individually housed in stainless steel cages. The environmental parameters were meticulously controlled, maintaining an ambient temperature of 23 ± 2 °C alongside relative humidity levels of 55–60%. A consistent 12-hour light/dark photoperiod cycle was sustained throughout the study. All animals had unrestricted access to standard laboratory diet and water throughout the experimental period.

#### Diet formulation

The experimental diet provided to the rats was a well-balanced formulation prepared according to the specifications established by Reeves et al.^14^. The compositional breakdown of the diet included 12% protein derived from casein, 10% corn oil, 10% sucrose, 58.5% corn starch, 5% cellulose, 3.5% AIN-93 salt mixture, and 1% AIN-93 vitamin mixture.

#### Ethical considerations

The experimental protocol received formal approval from the Institutional Animal Care and Use Committee of the Agriculture Research Centre (ARC-IACUC), with the assigned ethical approval number ARC-NRC-132-25. All procedures conducted during this investigation were performed in strict compliance with the National Institutes of Health guidelines pertaining to the care and appropriate use of laboratory animals^15^. Furthermore, the experiments were executed in accordance with all applicable guidelines and regulations, including the ARRIVE guidelines, the U.K. Animals Act 1986 along with its associated guidelines, and relevant EU Directives.

### Experimental methodology

#### Experimental design and animal grouping

After completion of the two-week acclimatization phase, a total of 32 rats were randomly assigned to four experimental groups, with each group containing eight animals (*n* = 8 per group) as follows:

- ***Group 1 (Normal control)***: Animals in this group received the balanced diet al.ongside daily oral administration of corn oil (functioning as the vehicle for lycopene) delivered via gastric gavage, combined with daily intraperitoneal injections of 0.9% normal saline (functioning as the vehicle for dexamethasone). These treatments were continued for 21 days, and this group served as the control reference.
- ***Group 2 (Dexamethasone (Dex))***: Animals in this group received the balanced diet with daily intraperitoneal injections of dexamethasone (2 mg/kg/day) and daily oral administration of corn oil (vehicle for lycopene) via gastric gavage for 21 consecutive days^16,17^.
- ***Group 3 (Lycopene-treated group)***: Animals in this group received the balanced diet with daily oral administration of lycopene (10 mg/kg/day dissolved in corn oil) via gastric gavage and daily intraperitoneal injections of 0.9% normal saline (vehicle for dexamethasone) for 21 consecutive days^18,19^.
- ***Group 4 (Dexamethasone + Lycopene***,*** Dex+Lyco)***: Animals in this group received the balanced diet with daily intraperitoneal injections of dexamethasone (2 mg/kg/day) and daily oral administration of lycopene (10 mg/kg/day dissolved in corn oil) via gastric gavage for 21 consecutive days.

Throughout the experimental period, all animals maintained continuous access to tap water and their respective dietary formulations. Both food and water supplies were replenished on a daily basis. At the end of the study, final body weight, cumulative food intake, and body weight gainwere evaluated. The feed efficiency ratio was subsequently calculated as the quotient of weight gain divided by total food intake. Additionally, the relative organs weight was determined using the following formula: (organ weight / final body weight) × 100, according to the methodology described by Chapman et al.^20^.

#### Assessment of depression-like behaviors

Behavioral evaluations were initiated 24 h after the final treatment administration. To eliminate potential olfactory interference, all testing apparatuses were thoroughly cleaned with 70% ethanol between trials and subsequently allowed to dry for approximately one minute. A trained observer who remained blinded to the treatment allocations manually performed all behavioral assessments throughout the experiments.

A. **Forced Swim Test (FST)**

The forced swim test was conducted after the 21-day intervention period. Animals were individually placed into transparent glass cylinders (20 cm diameter, 41 cm height) filled with water at 25 ± 1 °C to a depth of 30 cm, preventing the animals from touching the bottom. An initial 15-minute acclimatization session was conducted, after which rats were dried, warmed, and returned to their home cages. Twenty-four hours later, a 5-minute test session was performed under identical conditions, with cylinders cleaned between subjects. Three behavioral parameters were quantified: immobility time (passive floating with minimal movements to maintain buoyancy), swimming time (active horizontal movements using forepaws), and climbing time (vigorous vertical movements against the cylinder walls using all limbs)^21,22^.

B. **Tail Suspension Test (TST)**

For the tail suspension test, rats were suspended 28 ± 2 cm above the floor by securely fastening their tails at a point 2 cm from the tip. Immobility time was automatically recorded during the 6-minute test session^23,24^. Immobility was defined as complete cessation of all active movements, including limb swinging and body twisting.

#### Blood and brain tissue collection

Upon completion of the behavioral assessments, animals underwent a 12-hour fasting period prior to terminal procedures. Anesthesia was induced using a 1:1 combination of xylazine and ketamine administered intraperitoneally at a volume of 0.15 mL per 100 g body weight. Blood samples were collected from the retro-orbital venous plexus into two clean, dry tubes, with one tube containing EDTA for subsequent hematological evaluation, including hemoglobin determination, total white blood cell count, and differential leukocyte count. The hematological studies, including the complete leukogram, were performed using an automatic cell counter according to the methodology described by Thrall et al.^25^.

Serum was separated by centrifugation at 3000 rpm for 15 min at 4 °C using a Laborezentrifugen 2k15 centrifuge (Sigma, Germany), after which the samples were stored at -20 °C for subsequent biochemical analyses. Following blood collection, rats were euthanized via cervical dislocation.

Whole brains were rapidly excised and carefully dissected into two distinct regions: the prefrontal cortex and the hippocampus. The prefrontal cortex was identified as the anterior portion of the frontal lobe, localized rostral to the bregma and bounded dorsally by the rhinal fissure, and was dissected bilaterally using standard anatomical landmarks. Each individual brain region was homogenized using a mechanical homogenizer (MPW-120, BitLab Medical Instruments, Poland) at a concentration of 10% weight/volume in ice-cold phosphate buffer maintained at pH 7.4. The homogenates were subsequently centrifuged at 4000 rpm for 10 min at 4 °C using a cooling centrifuge (Laboratory Centrifuge, 2K15, Sigma Co., Germany) according to the protocol established by Essa et al.^26^. The resulting supernatants were collected and stored at -20 °C for subsequent analyses of oxidative and antioxidant markers, neurotransmitter quantification, and cytokine profiling.

#### Assessment of Interleukin-4 (IL-4), Interleukin-6 (IL-6), Tumor Necrosis Factor-alpha (TNF-α), and Caspase-3

Concentrations of TNF-α, IL-6, and caspase-3 in brain homogenates were quantified using rat-specific enzyme-linked immunosorbent assay (ELISA) kits obtained from Sunlong Biotechnology Co., LTD (Hangzhou, China), with catalog numbers SL0722Ra, SL0411Ra, and SL0152Ra, respectively. Serum levels of TNF-α and IL-4 were similarly quantified using rat-specific ELISA kits (catalog numbers SL0722Ra and SL0036Ra, respectively) from the same manufacturer, following the protocols provided by the manufacturer.

#### Assessment of Acetylcholinesterase (AChE), Gamma-Aminobutyric Acid (GABA), and Brain-Derived Neurotrophic Factor (BDNF)

AChE activity, GABA concentrations, and BDNF levels in brain homogenates were measured using rat-specific ELISA kits (catalog numbers SL0027Ra, SL0299Ra, and SL1207Ra, respectively) procured from Sunlong Biotechnology Co., LTD (Hangzhou, China). All assays were performed strictly according to the manufacturer’s provided protocols.

#### Evaluation of dopamine and serotonin in brain homogenates

Dopamine (catalog number SL0243Ra) and serotonin (catalog number SL1046Ra) concentrations in brain homogenates were determined using rat-specific ELISA kits obtained from Sunlong Biotechnology Co., LTD (Hangzhou, China), with all procedures conducted in accordance with the manufacturer’s instructions.

#### Assessment of oxidative and antioxidant parameters

Oxidative status in both brain tissue and serum was evaluated by quantifying the levels of malondialdehyde (MDA, serving as a lipid peroxidation marker), reduced glutathione (GSH), superoxide dismutase (SOD), catalase (CAT), and nitric oxide (NO). Spectrophotometric analyses were performed according to established methodologies: MDA was assessed following the method of Nair and Turner^27^; GSH was measured according to Jollow et al.^28^; SOD activity was determined using the procedure described by Sun et al.^29^; CAT activity was evaluated following the protocol of Luck^30^; and NO levels were quantified according to the method of Montgomery and Dymock^31^. All measurements were conducted using commercial colorimetric kits in accordance with the manufacturers’ protocols. Analyses for MDA, GSH, SOD, CAT, and NO were performed using kits obtained from Spectrum Biodiagnostics (Cairo, Egypt) with catalog numbers MD 25 29, GR 25 11, SD 25 21, CA 25 17, and NO 25 33, respectively. Optical density measurements for all parameters were recorded using a Shimadzu UV-2401 PC spectrophotometer (Australia), with all analyses performed precisely as specified in the respective kit instructions.

#### Assessment of cluster of differentiation 4 (CD4), cluster of differentiation 8 (CD8), and interferon-gamma (IFN-γ)

Serum levels of CD4, CD8, and IFN-γ were evaluated using rat-specific enzyme-linked immunosorbent assay (ELISA) kits obtained from Sunlong Biotechnology Co., LTD (Hangzhou, China), bearing catalog numbers SL1201Ra, SL0172Ra, and EL0015Ra, respectively. All procedures were conducted following the guidance provided by the manufacturer.

#### Biochemical parameter analysis

Fasting blood glucose levels were quantified using an enzymatic colorimetric assay according to the method described by Trinder^32^. Insulin concentrations were measured using a rat-specific ELISA kit (catalog number SL0373Ra) from Sunlong Biotechnology Co., LTD (Hangzhou, China). Insulin resistance was subsequently calculated using the homeostasis model assessment-estimated insulin resistance (HOMA-IR) index according to the following formula: HOMA-IR = [fasting glucose (mg/dL) × fasting insulin (µU/mL)] / 405, as established by Matthews et al.^33^.

Liver and kidney function biomarkers were assessed by measuring serum concentrations of aspartate aminotransferase (AST) and alanine aminotransferase (ALT) using the method of Reitman and Frankel^34^. Urea levels were determined according to the procedure described by Fawcett and Scott^35^, while creatinine concentrations were measured following the method of Larsen^36^. Total protein levels were assessed according to Richard^37^, and albumin concentrations were determined following the methodology of Doumas et al.^38^. These assays were performed using commercially available kits obtained from Spectrum Diagnostic (MDSS GmbH, Hannover, Germany) bearing catalog numbers 292 004, 320 002, 234 001, 310 001, and 210 001, respectively.

Globulin levels were calculated using the following equation: globulin levels = total protein levels − albumin levels, with fibrinogen amounts being disregarded as referenced by Burtis et al.^39^. The albumin-to-globulin (Alb/Glo) ratio was subsequently calculated from these values.

#### Statistical analysis

All results were analyzed using a one-way analysis of variance (ANOVA) followed by Tukey’s post-hoc test for multiple comparisons, utilizing SPSS software (Version 25) to identify significant differences between experimental groups. Data are presented throughout as means ± standard error (SE). Statistically significant differences between groups are indicated by different uppercase letters, with significance defined at *p* ≤ 0.05.

## Results

### Effects on depression-like behaviors

**Fig. 1:**
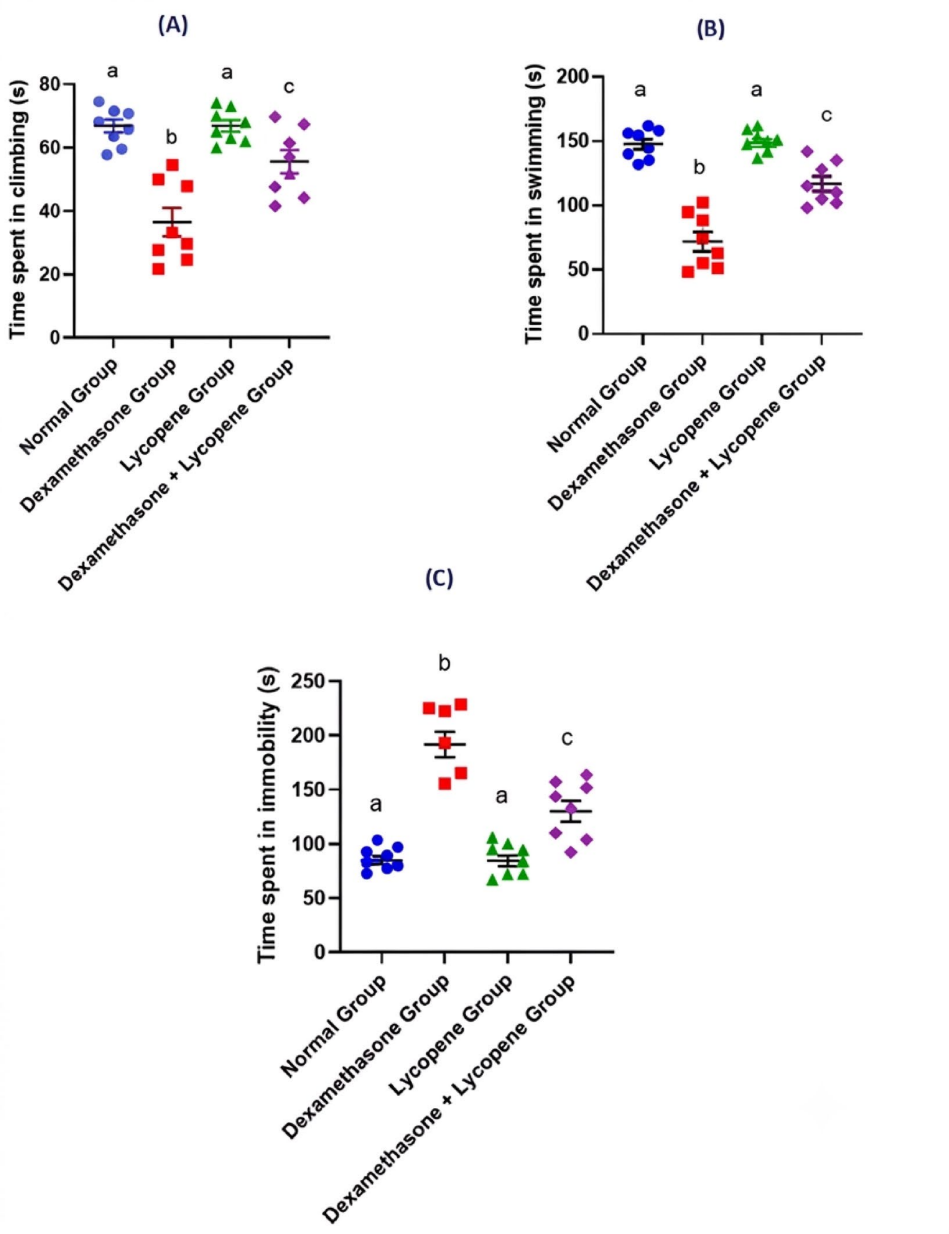
Effects of dexamethasone and lycopene co-treatment on depression-like behaviors in rats. **(A)** Forced Swim Test (FST): (A1) Time spent climbing (s), (A2) Time spent swimming (s), (A3) Immobility time (s) recorded during the FST as described in the Materials and Methods section. **(B)** Tail Suspension Test (TST): (B1) Immobility time (s) recorded during the TST. Data are presented as mean ± SE with individual animal data points superimposed (*n* = 8 rats per group). Statistical analysis was performed using one-way ANOVA followed by Tukey’s post-hoc test evaluating all pairwise comparisons between the four groups. Bars bearing different letters (a, b, c) indicate statistically significant differences between the respective groups at *p* < 0.05, whereas bars sharing the same letter are not significantly different.

**Fig. 2:**
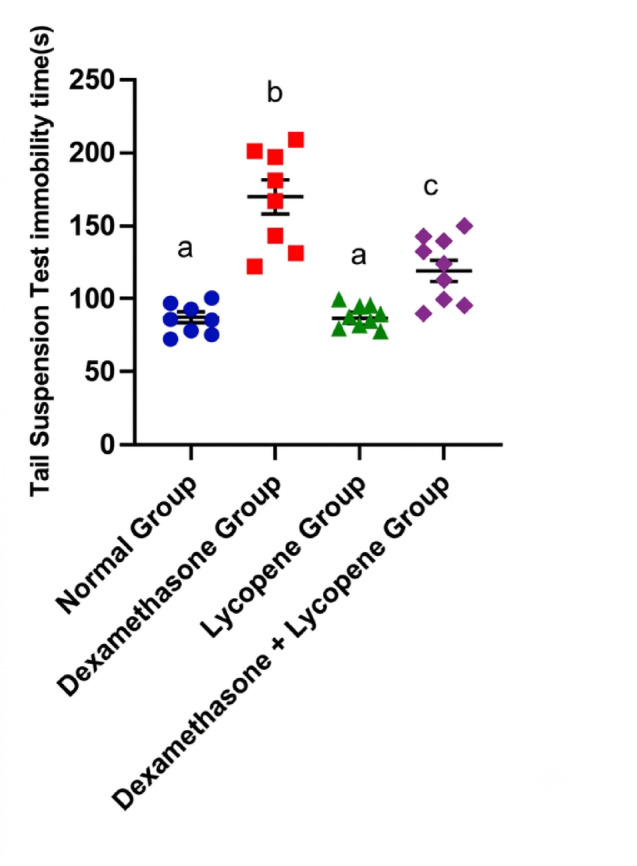
Effects of dexamethasone and lycopene co-treatment on immobility time (s) in the tail suspension test (TST). Data are presented as mean ± SE with individual animal data points superimposed (*n* = 8 rats per group). Statistical analysis was performed using one-way ANOVA followed by Tukey’s post-hoc test evaluating all pairwise comparisons between the four groups. Bars bearing different letters (a, b, c) indicate statistically significant differences between the respective groups at *p* < 0.05, whereas bars sharing the same letter are not significantly different.

**Forced Swim Test (FST**): Dexamethasone administration induced a depressive-like behavioral state, evidenced by approximately 2.5-fold increase in immobility time and corresponding reductions of approximately 65% and 55% in swimming and climbing times, respectively, compared to the normal control. Concomitant treatment with lycopene ameliorated these behavioral alterations, reducing dexamethasone-elevated immobility by approximately 30% while increasing both swimming and climbing times by roughly 90% and 70%, respectively, relative to the positive control group (Fig. 1A1, A2, A3).

#### Tail Suspension Test (TST):

Dexamethasone administration induced a depressive-like behavioral effect, evidenced by an approximate 2-fold increase in immobility time compared to the normal control. Co-treatment with lycopene mitigated this response, reducing the dexamethasone-elevated immobility time by approximately 30% relative to the positive control group (Fig. 2).

**Table 1:** Nutritional parameters, organ weights, and relative organ indices among the different experimental groups.

Parameters	Normal Control	Dexamethasone (Dex)	Lycopene Only	Dex + Lycopene
Initial weight (g)	215.42 ± 5.32 ^a^	216.18 ± 4.88 ^a^	214.95 ± 5.12 ^a^	215.80 ± 4.95 ^a^
Final weight (g)	342.15 ± 8.12 ^a^	255.64 ± 9.45 ^b^	338.40 ± 7.95 ^a^	298.22 ± 8.64 ^c^
Body weight gain (g)	126.73 ± 6.45 ^a^	39.46 ± 5.12 ^b^	123.45 ± 6.22 ^a^	82.42 ± 5.88 ^c^
Total Food intake (g)	445.80 ± 12.35 ^a^	348.12 ± 15.64 ^b^	442.15 ± 11.88 ^a^	395.45 ± 14.12 ^c^
Feed efficiency ratio	0.284 ± 0.01 ^a^	0.113 ± 0.02 ^b^	0.279 ± 0.01 ^a^	0.208 ± 0.02 ^c^
Brain weight (g)	1.96 ± 0.04 ^a^	1.78 ± 0.05 ^b^	1.94 ± 0.04 ^a^	1.88 ± 0.03 ^c^
Brain index (%)	0.573 ± 0.02 ^a^	0.696 ± 0.03 ^b^	0.573 ± 0.02 ^a^	0.630 ± 0.03 ^c^
Spleen weight (g)	0.74 ± 0.05 ^a^	0.38 ± 0.04 ^b^	0.72 ± 0.04 ^a^	0.55 ± 0.05 ^c^
Spleen index (%)	0.216 ± 0.01 ^a^	0.149 ± 0.02 ^b^	0.213 ± 0.01 ^a^	0.184 ± 0.02 ^c^
Thymus weight (g)	0.51 ± 0.03 ^a^	0.16 ± 0.02 ^b^	0.49 ± 0.03 ^a^	0.32 ± 0.04 ^c^
Thymus index (%)	0.149 ± 0.01 ^a^	0.063 ± 0.01 ^b^	0.145 ± 0.01 ^a^	0.107 ± 0.02 ^c^

All data are presented as Mean ± SE. Statistical significance was assessed using one-way ANOVA followed by Tukey’s post-hoc test. Superscript letters denote statistical differences within each row: values sharing identical letters (e.g., a or b) are not significantly different (*p* > 0.05). Distinct letters indicate a statistically significant difference (*p* < 0.05) between the groups.

Initial body weights showed no significant differences across all groups, confirming successful randomization. However, final body weight was reduced by 0.75-fold in the dexamethasone group relative to normal controls. Body weight gain exhibited a dramatic 0.31-fold reduction, accompanied by a 0.78-fold decrease in total food intake and a 0.40-fold reduction in feed efficiency ratio compared to normal values. Absolute brain weight decreased by 0.91-fold; however, the relative brain index showed an increase of 1.21-fold, indicating relative brain sparing despite overall growth suppression. Lymphoid organs were severely affected, with absolute spleen and thymus weights exhibiting marked reductions of 0.51-fold and 0.31-fold, respectively, compared to normal controls. The relative spleen and thymus indices were diminished by 0.69-fold and 0.42-fold, respectively, confirming profound immunosuppression. Lycopene administration alone maintained all parameters at levels statistically indistinguishable from normal controls. In the dexamethasone + lycopene co-treated group, amelioration of dexamethasone-induced growth retardation and organ atrophy was observed across all parameters. Specifically, final body weight increased by 16.7%, body weight gain increased by 108.9%, total food intake increased by 13.6%, and feed efficiency ratio increased by 84.1% compared to the dexamethasone-only group. Absolute brain weight increased by 5.6%, while the brain index decreased by 9.5%, approaching normal values. Absolute spleen and thymus weights showed substantial recovery, increasing by 44.7% and 100.0%, respectively, with corresponding relative indices improving by 23.5% and 69.8% compared to dexamethasone-treated animals (Table 1).

**Table 2:** Effects of lycopene on dexamethasone-induced oxidative stress and inflammation in rat prefrontal cortex and hippocampus.

Brain Tissues	Parameters	Normal Control	Dexamethasone (Dex)	Lycopene	Dex + Lycopene
Prefrontal Cortex	**MDA (nmol/g)**	5.56 ± 0.91ᵃ	11.51 ± 1.76ᵇ	4.95 ± 0.98ᵃ	7.85 ± 1.45ᶜ
	**SOD (U/g)**	75.10 ± 0.60ᵃ	52.80 ± 1.15^b^	76.50 ± 0.65ᵃ	68.50 ± 0.90^c^
	**NO (nmol/g)**	49.52 ± 0.86ᵃ	58.14 ± 1.12ᵇ	41.75 ± 0.65ᵃ	45.50 ± 0.88ᶜ
	**GSH (µmol/g)**	26.28 ± 0.69ᵃ	14.17 ± 0.45ᵇ	27.15 ± 0.58ᵃ	18.90 ± 0.51ᶜ
	**Catalase (U/g)**	55.80 ± 2.7ᵃ	28.51 ± 2.1ᵇ	53.58 ± 2.8ᵃ	43.03 ± 2.5ᶜ
	**IL-6 (pg/g)**	8.80 ± 0.70ᵃ	17.50 ± 1.10^b^	9.15 ± 0.75ᵃ	12.90 ± 0.85^c^
	**TNF-alpha (pg/g)**	12.78 ± 0.89ᵃ	25.30 ± 2.25ᵇ	10.15 ± 1.05ᵃ	16.50 ± 1.55ᶜ
	**Caspase-3 (ng/g)**	6.05 ± 0.47ᵃ	19.5 ± 1.15ᵇ	7.21 ± 0.39ᵃ	11.5 ± 0.85ᶜ
Hippocampus	**MDA (nmol/g)**	3.54 ± 0.83ᵃ	8.50 ± 1.55ᵇ	3.25 ± 0.92ᵃ	5.10 ± 1.25ᶜ
	**SOD (U/g)**	93.18 ± 0.73ᵃ	48.50 ± 0.95ᵇ	95.05 ± 0.69ᵃ	65.80 ± 0.85ᶜ
	**NO (nmol/g)**	37.48 ± 0.58ᵃ	55.10 ± 0.98ᵇ	37.95 ± 0.51ᵃ	43.20 ± 0.75ᶜ
	**GSH (µmol/g)**	21.72 ± 0.69ᵃ	11.50 ± 0.35ᵇ	19.85 ± 0.48ᵃ	16.10 ± 0.42ᶜ
	**Catalase (U/g)**	30.28 ± 2.9ᵃ	20.1 ± 1.5ᵇ	38.50 ± 2.1ᵃ	34.0 ± 1.9ᶜ
	**IL-6 (pg/g)**	6.65 ± 0.66ᵃ	20.10 ± 1.75ᵇ	7.35 ± 0.61ᵃ	11.50 ± 1.05ᶜ
	**TNF-alpha (pg/g)**	8.59 ± 1.19ᵃ	23.50 ± 2.10ᵇ	9.45 ± 1.45ᵃ	15.10 ± 1.80ᶜ
	**Caspase-3 (ng/g)**	11.82 ± 0.39ᵃ	21.0 ± 1.25ᵇ	8.90 ± 0.48ᵃ	12.5 ± 0.95ᶜ

All data are presented as Mean ± SE. Statistical significance was assessed using one-way ANOVA followed by Tukey’s post-hoc test. Superscript letters denote statistical differences within each row: values sharing identical letters (e.g., a or b) are not significantly different (*p* > 0.05). Distinct letters indicate a statistically significant difference (*p* < 0.05) between the groups.

Dexamethasone treatment induced a consistent pattern of cerebral alteration in both the prefrontal cortex and hippocampus, characterized by elevated oxidative stress and inflammation. Compared to the normal control, the Dex group exhibited an approximate 2.1-fold increase in lipid peroxidation (MDA) and a significant depletion of key antioxidants: GSH decreased by 46% in the prefrontal cortex and 48% in the hippocampus, while catalase activity was nearly halved. Pro-inflammatory cytokines (IL-6, TNF-α) and apoptotic caspase-3 were also markedly elevated. Co-administration of lycopene mitigated these disturbances. It reduced MDA levels by approximately 32% in the prefrontal cortex and 40% in the hippocampus, increased GSH by 33% in the prefrontal cortex and 40% in the hippocampus, and elevated catalase activity by approximately 51% and 69%, respectively, compared to the Dex-only group. Furthermore, lycopene treatment lowered IL-6, TNF-α, and caspase-3, demonstrating a substantial protective effect across both brain regions. The lycopene-only group showed no significant differences from the normal control across all parameters (Table 2).

**Table 3:** Effect of dexamethasone-induced neurochemical alterations and lycopene treatment on BDNF, AChE, GABA, serotonin, and dopamine levels in the Prefrontal cortex and hippocampus of rats.

Brain Tissues	Parameters	Normal Control	Dexamethasone (Dex)	Lycopene	Dex + Lycopene
Prefrontal Cortex	**BDNF** **(µg/g tissue)**	65.51 ± 3.87ᵃ	48.15 ± 6.98ᵇ	70.72 ± 4.04ᵃ	63.47 ± 4.50ᶜ
	**AChE** **(U/g tissue)**	258.74 ± 4.93ᵃ	354.74 ± 10.22ᵇ	259.01 ± 5.62ᵃ	292.21 ± 6.76ᶜ
	**GABA** **(µg/g tissue)**	40.27 ± 2.98ᵃ	30.91 ± 7.18ᵇ	45.14 ± 3.69ᵃ	39.82 ± 4.66ᶜ
	**Serotonin (ng/g tissue)**	418.04 ± 7.06ᵃ	316.80 ± 8.99ᵇ	425.48 ± 6.86ᵃ	425.59 ± 5.34ᶜ
	**Dopamine (ng/g tissue)**	392.03 ± 5.71ᵃ	336.00 ± 7.80ᵇ	395.94 ± 5.63ᵃ	443.05 ± 4.69ᶜ
Hippocampus	**BDNF** **(µg/g tissue)**	78.33 ± 4.12ᵃ	56.12 ± 7.36ᵇ	84.58 ± 3.85ᵃ	75.49 ± 5.13ᶜ
	**AChE** **(U/g tissue)**	204.69 ± 3.88ᵃ	258.90 ± 7.06ᵇ	199.55 ± 3.77ᵃ	217.28 ± 4.89ᶜ
	**GABA** **(µg/g tissue)**	50.61 ± 2.84ᵃ	33.54 ± 5.65ᵇ	53.02 ± 3.11ᵃ	46.44 ± 3.71ᶜ
	**Serotonin (ng/g tissue)**	424.80 ± 5.47ᵃ	271.90 ± 9.25ᵇ	426.00 ± 5.91ᵃ	350.20 ± 5.70ᶜ
	**Dopamine (ng/g tissue)**	401.31 ± 5.78ᵃ	226.91 ± 7.31ᵇ	405.08 ± 5.57ᵃ	306.30 ± 5.54ᶜ

All data are presented as Mean ± SE. Statistical significance was assessed using one-way ANOVA followed by Tukey’s post-hoc test. Superscript letters denote statistical differences within each row: values sharing identical letters (e.g., a or b) are not significantly different (*p* > 0.05). Distinct letters indicate a statistically significant difference (*p* < 0.05) between the groups.

In the prefrontal cortex, dexamethasone administration induced neurochemical disruptions compared to normal control values. Specifically, BDNF levels were reduced by approximately 0.74-fold, while AChE activity showed a marked 1.37-fold increase. GABA, serotonin, and dopamine concentrations exhibited reductions of 0.77-fold, 0.76-fold, and 0.86-fold, respectively, relative to normal controls. Co-administration of lycopene showed improvements relative to the dexamethasone-only group, with BDNF increasing by 31.8%, AChE decreasing by 17.6%, GABA increasing by 28.8%, serotonin increasing by 34.4%, and dopamine increasing by 31.9%.

In the hippocampus, a more pronounced neurochemical disruption was evident. Dexamethasone administration reduced BDNF by 0.72-fold, increased AChE by 1.26-fold, and decreased GABA by 0.66-fold. Hippocampal serotonin and dopamine were markedly reduced by 0.64-fold and 0.57-fold, respectively, compared to normal controls. The combined dexamethasone and lycopene treatment ameliorated the dexamethasone-induced alterations, demonstrating increases in BDNF (34.5%), GABA (38.5%), serotonin (28.8%), and dopamine (35.0%), alongside a reduction in AChE activity (16.1%) when compared to the dexamethasone-only group. The lycopene-only group showed no significant differences from the normal control across all parameters (Table 3).

**Fig. 3:**
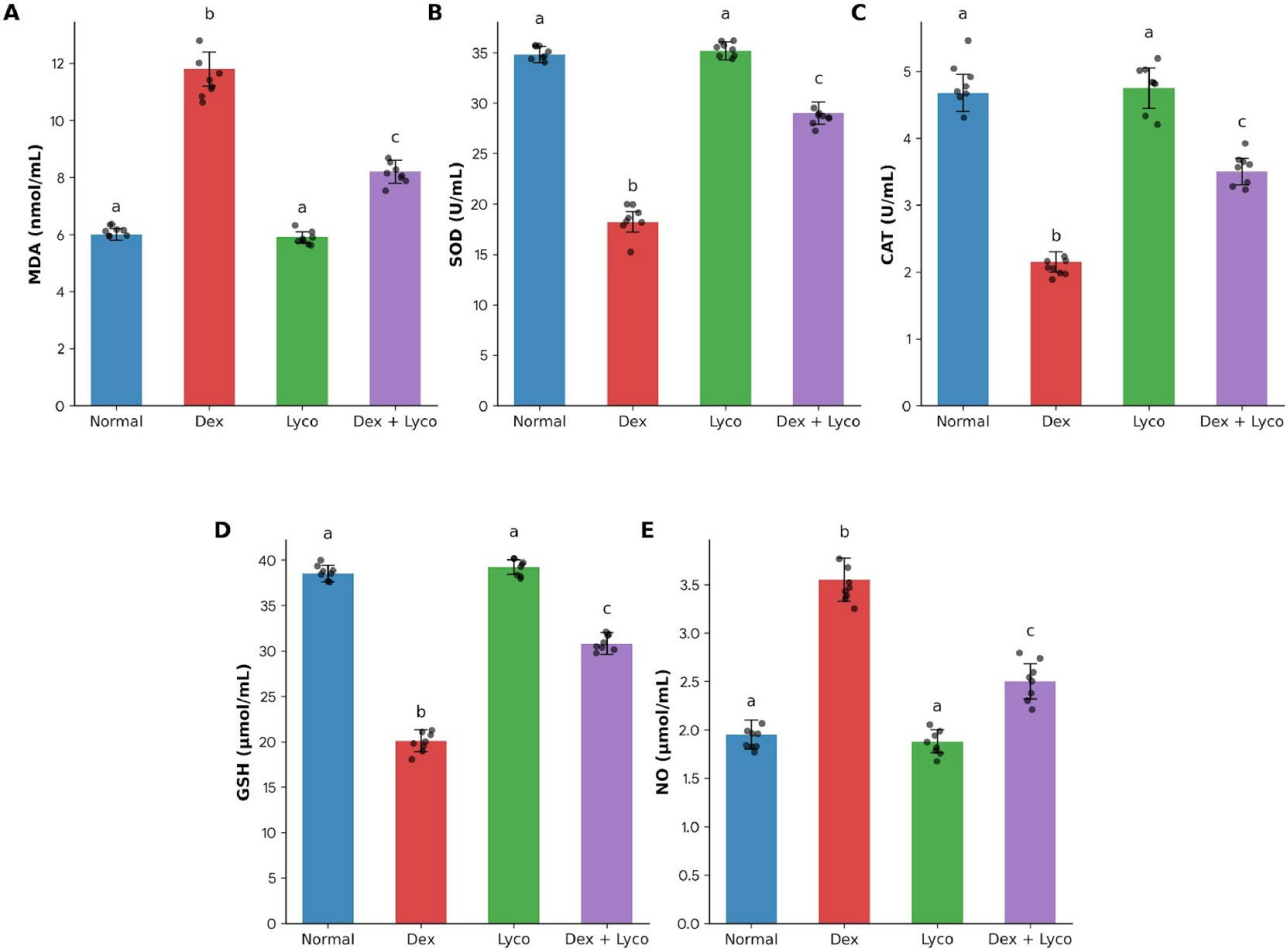
Serum oxidative stress markers in rats treated with dexamethasone and lycopene co-administration. Bar graphs representing **(A)** malondialdehyde (MDA), **(B)** superoxide dismutase (SOD), **(C)** catalase (CAT), **(D)** reduced glutathione (GSH), and **(E)** nitric oxide (NO) levels across the four experimental groups: normal control, dexamethasone (Dex), lycopene (Lyco), and combination (Dex+Lyco). Data are expressed as mean ± SE with individual animal data points superimposed (*n* = 8 rats per group). Statistical analysis was performed using one-way ANOVA followed by Tukey’s post-hoc test evaluating all pairwise comparisons between the four groups. Bars bearing different letters (a, b, c) indicate statistically significant differences between the respective groups at *p* < 0.05, whereas bars sharing the same letter are not significantly different.

Administration of dexamethasone induced oxidative stress compared to normal control values, as evidenced by marked alterations in all measured parameters. Lipid peroxidation, reflected by MDA levels, exhibited a substantial 1.98-fold increase (98.5% elevation) in the dexamethasone group relative to normal controls. Concurrently, the antioxidant defense mechanisms were compromised, with SOD activity declining by 0.52-fold, CAT activity decreasing by 0.46-fold, and GSH levels diminishing by 0.52-fold compared to normal values. Nitric oxide levels demonstrated a 1.82-fold increase in the dexamethasone-treated group relative to normal controls. Treatment with lycopene alone maintained all parameters at levels statistically indistinguishable from normal controls, indicating no inherent pro-oxidant effects. In the dexamethasone + lycopene co-treated group, amelioration of dexamethasone-induced oxidative damage was observed across all parameters. Specifically, MDA levels decreased by 31.2%, while SOD, CAT, and GSH increased by 58.4%, 62.8%, and 53.2%, respectively, compared to the dexamethasone-only group. Furthermore, NO levels were reduced by 29.6% in the combination group relative to dexamethasone-treated animals (Fig. 3A–E).

**Fig. 4:**
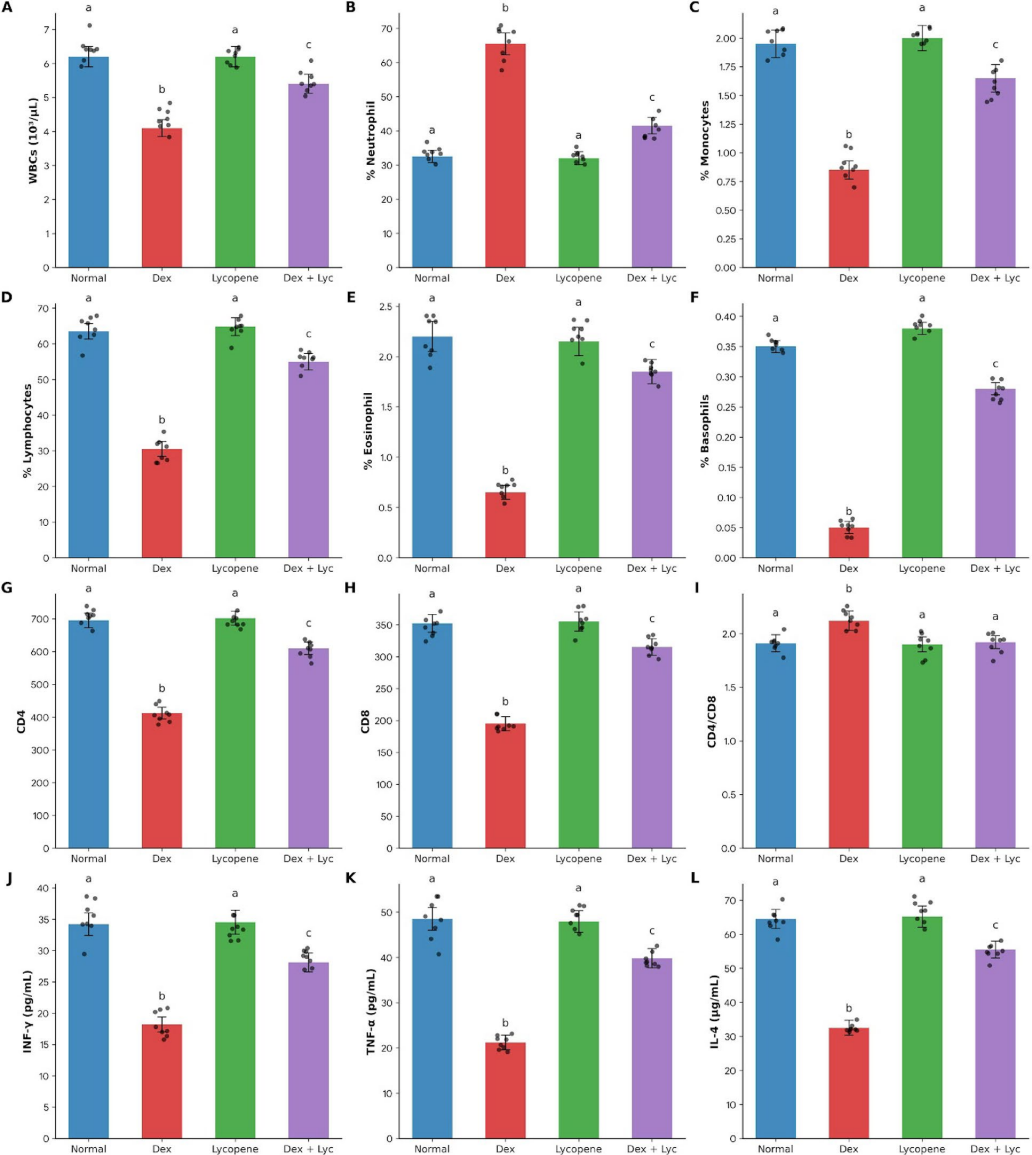
Effects of dexamethasone-induced immunosuppression and lycopene treatment on hematological parameters, lymphocyte subsets, and cytokine profiles in rats. Bar graphs representing **(A)** total leukocyte count (WBC, 10³/µL), **(B)** neutrophil percentage (%), **(C)** monocyte percentage (%), **(D)** lymphocyte percentage (%), **(E)** eosinophil percentage (%), **(F)** basophil percentage (%), **(G)** CD4 concentration (pg/mL), **(H)** CD8 concentration (pg/mL), **(I)** CD4/CD8 ratio, **(J)** interferon-gamma (IFN-γ, pg/mL), **(K)** tumor necrosis factor-alpha (TNF-α, pg/mL), and **(L)** interleukin-4 (IL-4, pg/mL) across the four experimental groups. Data are expressed as mean ± SE with individual animal data points superimposed (*n* = 8 rats per group). Statistical analysis was performed using one-way ANOVA followed by Tukey’s post-hoc test evaluating all pairwise comparisons between the four groups. Bars bearing different letters (a, b, c, d) indicate statistically significant differences between the respective groups at *p* < 0.05, whereas bars sharing the same letter are not significantly different.

Dexamethasone administration induced profound immunosuppression, as evidenced by alterations across all hematological and immunological parameters compared to normal controls. Total leukocyte count exhibited a marked 50.6% reduction, accompanied by a shift in differential leukocyte percentages. Neutrophils demonstrated a substantial 103.0% elevation, while lymphocytes showed a striking 51.7% reduction relative to normal values. Monocytes, eosinophils, and basophils were diminished by 56.2%, 70.5%, and 85.7%, respectively. Lymphocyte subset analysis revealed reductions in CD4 (40.7%) and CD8 (44.9%) concentrations, with a concomitant increase in the CD4/CD8 ratio (7.7%) compared to normal controls. Cytokine profiling demonstrated immunosuppressive and pro-inflammatory alterations, with IFN-γ and IL-4 declining by 0.46-fold (53.7% decrease) and 0.47-fold (52.5% decrease), respectively, while TNF-α exhibited a 1.92-fold increase (92.3% elevation) relative to normal values. The lycopene-only group maintained all parameters at levels statistically indistinguishable from normal controls, confirming its immunological safety profile. In the dexamethasone + lycopene co-treated group, amelioration of dexamethasone-induced immunosuppression was observed across all parameters. Specifically, WBCs increased by 69.5%, neutrophils decreased by 37.4%, lymphocytes increased by 79.7%, monocytes increased by 94.1%, eosinophils increased by 184.6%, and basophils increased by 460.0% compared to the dexamethasone-only group. The CD4 and CD8 concentrations were elevated by 48.1% and 61.6%, respectively, while the CD4/CD8 ratio returned to a value (1.93) comparable to normal controls. Cytokine levels showed improvement, with IFN-γ and IL-4 increasing by 77.2% and 81.6%, respectively, and TNF- α decreasing by 57.3% compared to dexamethasone-treated animals (Fig. 4A–L).

**Fig. 5:**
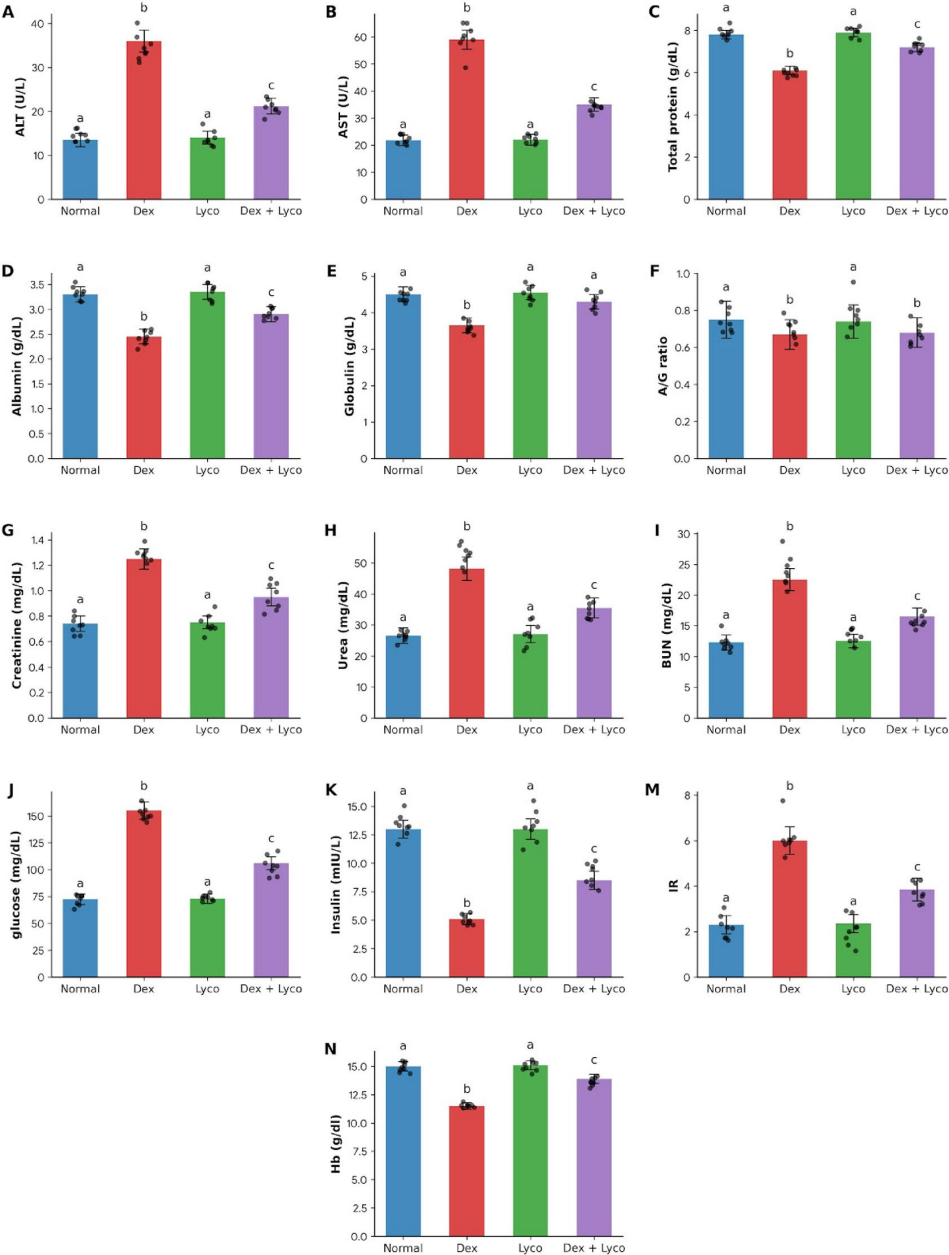
Effects of dexamethasone and lycopene treatment on hepatic function, renal function, and glucose homeostasis in rats. Bar graphs representing **(A)** alanine aminotransferase (ALT), **(B)** aspartate aminotransferase (AST), **(C)** total protein, **(D)** albumin, **(E)** globulin, **(F)** albumin-to-globulin (A/G) ratio, (G) creatinine, **(H)** urea, **(I)** blood urea nitrogen (BUN), **(J)** glucose, **(K)** insulin, **(L)** insulin resistance (HOMA-IR), and **(M)** hemoglobin (Hb). Data are expressed as mean ± SE with individual animal data points superimposed (*n* = 8 rats per group). Statistical analysis was performed using one-way ANOVA followed by Tukey’s post-hoc test evaluating all pairwise comparisons. Bars bearing different letters (a, b, c, d) indicate statistically significant differences between the respective groups at *p* < 0.05, whereas bars sharing the same letter are not significantly different.

Dexamethasone administration induced multi-organ metabolic disturbances compared to normal control values. Hepatic injury markers were markedly elevated, with ALT showing a 2.68-fold increase and AST demonstrating a 2.72-fold increase relative to normal controls. Renal function parameters were compromised, as evidenced by creatinine increasing 1.69-fold, urea rising 1.82-fold, and BUN increasing 1.82-fold compared to normal values. Glucose homeostasis was severely disrupted, with fasting glucose exhibiting a 2.14-fold increase while insulin declined by 0.40-fold, resulting in a 2.58-fold increase in insulin resistance (IR) index relative to normal controls. Hematological assessment revealed a 23.3% decrease in hemoglobin concentration. Protein profile analysis demonstrated reductions in total protein (21.4%), albumin (26.2%), and globulin (17.8%), with a concomitant decrease in the A/G ratio (10.7% reduction) compared to normal controls. Lycopene monotherapy maintained all parameters at levels statistically indistinguishable from normal controls, confirming its safety profile.

In the dexamethasone + lycopene co-treated group, amelioration of dexamethasone-induced metabolic disturbances was observed across all parameters. Specifically, ALT and AST decreased by 41.2% and 41.2%, respectively, compared to the dexamethasone-only group. Renal function markers showed improvement, with creatinine, urea, and BUN declining by 24.0%, 26.2%, and 26.5%, respectively. Glucose homeostasis was enhanced, with glucose decreasing by 31.9%, insulin increasing by 66.7%, and IR decreasing by 35.3% relative to dexamethasone-treated animals. Hemoglobin concentration increased by 21.0% compared to the dexamethasone group. Protein profile demonstrated restoration, with total protein, albumin, and globulin increasing by 17.2%, 18.4%, and 16.4%, respectively, compared to dexamethasone-only values. Notably, the A/G ratio in the co-treated group (0.68) showed improvement compared to the dexamethasone group (Fig. 5A–M).

## Discussion

This study provides robust evidence that lycopene, a potent dietary carotenoid, effectively mitigates the dual neurobehavioral and immunological pathologies induced by chronic dexamethasone (DEX) administration in a rat model. Our findings demonstrate that prophylactic lycopene supplementation (10 mg/kg/day) significantly ameliorates depression-like behaviors, counteracts oxidative stress and neuroinflammation, preserves neurochemical and neurotrophic homeostasis, and concurrently prevents peripheral immunosuppression and metabolic dysfunction. The Lycopene-only group exhibited no deviations from the Normal Control, affirming the safety profile of the chosen dose and indicating that the observed benefits in the DEX+Lycopene group stem from active protection rather than intrinsic stimulation. These findings position lycopene as a compelling multi-target adjuvant candidate for countering the significant iatrogenic burdens associated with long-term glucocorticoid therapy.

Chronic DEX administration produced pronounced behavioral despair, evidenced by increased immobility and reduced active coping in the forced swim and tail suspension tests. These behaviors are canonical indices of a depression-like state, reflecting impaired motivation and stress adaptability driven by sustained glucocorticoid receptor activation and disruption of hippocampal–prefrontal circuitry^40–42^. Lycopene co-treatment significantly reversed these deficits, restoring active coping behaviors. This recovery was strongly correlated with the normalization of critical neurotransmitter systems in the hippocampus and prefrontal cortex regions with high glucocorticoid receptor density and established vulnerability^43,44^. Lycopene prevented DEX-induced depletion of serotonin and dopamine, attenuated elevated acetylcholinesterase activity, and restored GABA concentrations. This comprehensive stabilization of monoaminergic, cholinergic, and GABAergic systems provides a coherent neurochemical basis for the observed antidepressant-like effect^45,46^ and aligns with emerging clinical evidence linking carotenoid intake to positive mental health outcomes^47,48^.

Our data strongly indicate that the reversal of neurobehavioral deficits is mechanistically rooted in lycopene’s capacity to alleviate oxidative and nitrosative stress in the brain. DEX administration triggered a significant redox imbalance, marked by elevated MDA and NO alongside depleted GSH, SOD, and catalase in both the hippocampus and prefrontal cortex. This environment promotes lipid peroxidation, protein dysfunction, and mitochondrial damage, ultimately compromising neuronal integrity. As a powerful lipophilic antioxidant, lycopene directly quenches reactive oxygen and nitrogen species and upregulates endogenous antioxidant defenses, as evidenced by a significant improvement in redox profile in the DEX+Lycopene group^49,50^.

A particularly intriguing finding of this study is the apparent paradox that chronic dexamethasone (DEX) administration a potent synthetic glucocorticoid with well-established immunosuppressive properties was associated with elevated pro-inflammatory cytokines (IL-6 and TNF-α) and increased caspase-3 activity in the prefrontal cortex and hippocampus. This seemingly contradictory observation is increasingly recognized in the literature and can be explained by several converging mechanisms.

First, chronic glucocorticoid exposure can induce a state of glucocorticoid resistance, wherein prolonged activation of glucocorticoid receptors (GRs) leads to receptor desensitization, reduced GR nuclear translocation, and impaired transrepression of pro-inflammatory transcription factors such as NF-κB and AP-1^51,52^. This resistance phenomenon has been documented in both peripheral immune cells and central nervous system (CNS) tissues, effectively converting glucocorticoids from anti-inflammatory to pro-inflammatory mediators under chronic exposure conditions^53^.

Second, chronic glucocorticoid exposure primes microglia the resident immune cells of the CNS to adopt a hyper-reactive, pro-inflammatory phenotype^54^. This ‘microglial priming’ phenomenon, mediated in part by glucocorticoid-induced upregulation of NLRP3 inflammasome components, results in exaggerated production of IL-1β, IL-6, and TNF-α upon subsequent immune challenges^53^. Importantly, this priming effect persists even in the presence of ongoing glucocorticoid exposure, creating a neuroinflammatory environment that paradoxically coexists with systemic immunosuppression^54^.

Third, glucocorticoids exert region-specific effects within the brain. While they are potently anti-inflammatory in peripheral tissues and certain brain regions, the hippocampus and prefrontal cortex both rich in glucocorticoid receptors exhibit differential sensitivity^55^. Chronic DEX exposure in these regions disrupts the delicate balance between GR and mineralocorticoid receptor (MR) signaling, favoring pro-inflammatory transcriptional programs^55^.

Fourth, the central and peripheral immune compartments are differentially regulated. While DEX potently suppresses peripheral adaptive immunity (as evidenced by our leukopenia and lymphopenia findings), the CNS innate immune response mediated by microglia and astrocytes exhibits a distinct, and in some cases opposing, response to chronic glucocorticoids^54,55^.

Concurrently, lycopene potently suppressed the DEX-induced surge in pro-inflammatory cytokines IL-6 and TNF-α. This anti-neuroinflammatory action is crucial, as these cytokines are known to inhibit neurogenesis, impair synaptic plasticity, and directly influence monoamine metabolism. The observed reduction in the apoptotic marker caspase-3 further demonstrates that lycopene’s antioxidant and anti-inflammatory effects converge to enhance neuronal survival. This multi-faceted protection against glucocorticoid neurotoxicity is supported by recent research demonstrating lycopene’s efficacy in alleviating depression-like behavior via the promotion of synaptic plasticity through the BDNF-TrkB pathway^56^.

While the antioxidant properties of lycopene are well-established and undoubtedly contribute to its neuroprotective effects, our data suggest a broader, multi-system mechanism of action that extends well beyond simple radical scavenging.

At the molecular level, lycopene exerts its pleiotropic effects through modulation of several key signaling pathways. First, lycopene activates the nuclear factor erythroid-2-related factor 2 (Nrf2) pathway, leading to the upregulation of endogenous antioxidant enzymes including superoxide dismutase, catalase, and glutathione peroxidase, thereby enhancing cellular resilience against oxidative challenge^57^. Second, lycopene inhibits NF-κB nuclear translocation, reducing the transcription of pro-inflammatory cytokines including IL-6 and TNF-α^58^. This dual Nrf2 activation and NF-κB inhibition represents a coordinated anti-oxidant and anti-inflammatory response that is more potent than either effect alone^57^.

Third, lycopene modulates microglial polarization, shifting the balance from pro-inflammatory M1 to anti-inflammatory M2 phenotypes, thereby reducing neuroinflammation while promoting tissue repair and neurotrophic support^58^. Fourth, lycopene activates the BDNF-TrkB pathway, promoting synaptic plasticity and neuronal survival a mechanism that directly links its molecular actions to the observed behavioral recovery^56^.

Fifth, lycopene preserves mitochondrial membrane potential and reduces cytochrome c release, thereby inhibiting the intrinsic apoptotic pathway and caspase-3 activation^59^. This anti-apoptotic effect, combined with its anti-inflammatory and neurotrophic actions, provides comprehensive protection against glucocorticoid-induced neurotoxicity.

Importantly, lycopene’s immunomodulatory effects are not limited to the CNS. By protecting immune cells from oxidative, apoptosis and modulating cytokine pathways, lycopene preserves peripheral immune homeostasis, mitigating the immunosuppressive effects while maintaining immune competence^60^. This integrated neuro-immune protection addressing both central neurotoxicity and peripheral immunosuppression is a unique feature of lycopene that distinguishes it from conventional agents with more limited mechanisms of action.

By modulating redox-sensitive transcription factors like NF-κB and Nrf2, lycopene simultaneously dampens inflammatory signaling and enhances cellular antioxidant capacity^61,62^. A central finding supporting this integrated mechanism is the lycopene-mediated restoration of brain-derived neurotrophic factor (BDNF). BDNF is a critical mediator of synaptic plasticity and neuronal survival, and its suppression is a hallmark of depression^63,64^. Lycopene’s ability to elevate BDNF, likely through reducing oxidative and inflammatory suppression of neurotrophin signaling^65^, directly links its molecular actions to functional neuroprotection and behavioral recovery, a mechanism supported by recent preclinical evidence^66^.

A significant finding of this study is lycopene’s capacity to preserve peripheral immune homeostasis in the face of potent glucocorticoid suppression. The DEX group exhibited classic iatrogenic immunosuppression characterized by leukopenia, lymphopenia, and alterations in circulating CD4 and CD8 protein levels. However, it is important to exercise caution in interpreting the CD4/CD8 ratio reported in this study.

The CD4 and CD8 concentrations were measured in serum using ELISA, which quantifies soluble CD4 and CD8 proteins rather than CD4⁺ and CD8⁺ lymphocyte populations. Soluble CD4 and CD8 levels reflect shed forms of these molecules that may result from variations in protein expression, receptor shedding, release into circulation, or other biological processes. Importantly, serum soluble CD4 and CD8 concentrations do not necessarily correlate directly with T-cell subset abundance, as they may also be influenced by T-cell activation status, cellular turnover, and proteolytic cleavage^67,68^.

Therefore, while the observed alterations in circulating CD4 and CD8 protein levels, together with the complete blood count and differential leukocyte analysis, provide valuable information about immune status, they should not be interpreted as direct estimates of CD4⁺ or CD8⁺ T-cell populations. The CD4/CD8 ratio calculated from ELISA-based measurements should be viewed as an indirect indicator of immune function rather than a definitive measure of T-cell subset abundance. Future studies incorporating flow cytometric immunophenotyping would be necessary to definitively characterize T-cell population dynamics.

Notwithstanding these methodological limitations, the consistent pattern of leukopenia, lymphopenia, and altered CD4/CD8 protein levels in the DEX group, together with their significant amelioration by lycopene co-treatment, provides compelling evidence for lycopene’s immunomodulatory effects. This immunomodulatory effect likely stems from protecting immune cells from oxidative, apoptosis and modulating cytokine pathways, as supported by the concurrent improvements in IFN-γ, IL-4, and TNF-α profiles^60^.

This systemic protection extended to metabolic and organ function. DEX-induced hyperglycemia, elevated insulin resistance (HOMA-IR), and increased markers of hepatic (ALT, AST) and renal (creatinine, urea) stress were all significantly attenuated by lycopene. These benefits align with lycopene’s well-documented role in metabolic syndrome and its associated pathways^69^. The protection of organ function likely stems from the same antioxidant principles observed in the brain, as demonstrated in earlier work showing lycopene’s activity against DEX-induced oxidative stress in hepatic and renal tissues^70^. This systemic metabolic and organoprotective role aligns with lycopene’s documented benefits in models of metabolic syndrome^71^ and underscores its holistic protective profile against the peripheral toxicities of glucocorticoids^40,72^.

Importantly, it should be acknowledged that the neuroprotective effects of lycopene observed in this study may not be exclusively attributable to direct actions within the central nervous system (CNS). Given the marked systemic and metabolic disturbances induced by dexamethasone including hyperglycemia, insulin resistance, hepatic and renal dysfunction, and peripheral immunosuppression it is plausible that at least part of the observed neuroprotection results from indirect systemic actions^73,74^. Improvements in peripheral metabolic homeostasis, such as reduced hyperglycemia and insulin resistance, may attenuate systemic inflammation and oxidative stress, thereby reducing the peripheral inflammatory signals that can cross the blood-brain barrier and exacerbate neuroinflammation^75^. Furthermore, the preservation of peripheral immune competence and the reduction of circulating pro-inflammatory cytokines (TNF-α, IL-6) by lycopene may limit the influx of inflammatory mediators into the brain, indirectly mitigating neuroinflammatory responses^44,54^. This bidirectional communication between the peripheral and central immune compartments the neuro-immune axis suggests that lycopene’s protective effects are likely mediated through both direct CNS actions (e.g., crossing the blood-brain barrier, modulating microglial function, activating BDNF-TrkB signaling) and indirect systemic actions (e.g., improving metabolic function, reducing peripheral inflammation, preserving immune homeostasis). We propose that these direct and indirect mechanisms act synergistically to provide comprehensive neuroprotection against glucocorticoid-induced neurotoxicity^44,62,73,74^.

The collective data support an integrative model wherein chronic DEX exposure disrupts redox homeostasis, activates neuroinflammatory and apoptotic cascades, suppresses neurotrophic and neurotransmitter systems, and induces systemic immunosuppression and metabolic dysfunction. Lycopene interrupts this pathological cascade through interlinked antioxidant, anti-inflammatory, anti-apoptotic, neurotrophic, and immunomodulatory mechanisms. Its primary actions create a stabilized milieu across neural and immune systems: by scavenging free radicals and inhibiting NF-κB-mediated cytokine production, lycopene preserves mitochondrial and cellular integrity. This prevents apoptotic activation (reducing caspase-3), fosters a milieu conducive to neurotrophic support (raising BDNF), and allows for normal neurotransmission and immune cell viability. The bidirectional communication of the neuro-immune axis suggests that ameliorating peripheral immunosuppression may further dampen systemic inflammatory signals that exacerbate neuroinflammation, creating a virtuous cycle of protection^44,62,60^.

It is important to acknowledge that while microglia are emphasized in the introduction as central mediators of stress-associated neuroinflammation, the present study did not include microglia-specific measurements such as immunohistochemical staining for Iba-1 or CD68, or flow cytometric analysis of microglial phenotypes. Therefore, the cellular source of the inflammatory alterations observed in our study whether microglia, astrocytes, neurons, or infiltrating peripheral immune cells cannot be definitively determined.

Nevertheless, the literature provides strong evidence that microglia are primary mediators of glucocorticoid-induced neuroinflammation. Chronic glucocorticoid exposure primes microglia to adopt a pro-inflammatory phenotype characterized by increased CD68 and MHCII expression and hyper-reactive responses to subsequent immune challenges^54^. Furthermore, microglia are rich in glucocorticoid receptors, rendering them particularly susceptible to glucocorticoid-induced transcriptional changes^54^. Astrocytes, which also express glucocorticoid receptors, may contribute to the observed cytokine elevations through altered gliotransmitter release and inflammatory signaling^54^.

While our tissue-level measurements cannot distinguish between these cellular sources, the consistent pattern of elevated IL-6, TNF-α, and caspase-3 across both the prefrontal cortex and hippocampus is consistent with a microglial-driven neuroinflammatory response, as microglia are the primary source of pro-inflammatory cytokines within the CNS^44^. However, we acknowledge this as a limitation and recommend that future studies incorporate microglia-specific markers and immunohistochemical analyses to definitively identify the cellular origin of the neuroinflammatory changes observed.

This study pioneers the concurrent examination of dexamethasone-induced immunosuppression and depression-like behaviors in rats, with lycopene’s prophylactic efficacy evaluated across behavioral, neurochemical, oxidative, inflammatory, immunological, and metabolic domains within a single integrated experimental framework. While previous investigations have examined individual components of this cascade focusing either on neurobehavioral effects or on splenic immunosuppression none have simultaneously characterized the multi-system toxicity of glucocorticoid excess and the pleiotropic protective effects of lycopene across all these interconnected physiological systems.

### Study limitations and future directions

Several limitations warrant consideration in interpreting these findings. First, the use of a single lycopene dose (10 mg/kg) precludes dose-response analysis and identification of optimal therapeutic windows. Future studies should explore multiple doses to establish dose-dependency and potential threshold effects for different outcome measures. Second, the exclusive use of male rats, while controlling for estrous cycle variability, limits generalizability to females, who exhibit higher rates of stress-related mood disorders and may show differential glucocorticoid sensitivity. Future investigations should include both sexes to examine potential dimorphisms in response, particularly given known sex differences in HPA axis function and glucocorticoid receptor expression. Third, as noted above, the absence of microglia-specific measurements limits our ability to definitively identify the cellular source of the neuroinflammatory changes observed. Future studies incorporating immunohistochemical staining for microglial markers (Iba-1, CD68), astrocytic markers (GFAP), and neuronal markers (NeuN) would help elucidate the cellular origin of the observed inflammatory and apoptotic changes. Fourth, the measurement of circulating CD4 and CD8 proteins by ELISA, rather than flow cytometric immunophenotyping, limits our ability to definitively characterize T-cell population dynamics. Future studies should employ flow cytometry to quantify CD4⁺ and CD8⁺ lymphocyte populations directly. Fifth, while the present findings demonstrate prophylactic efficacy, future studies should investigate whether lycopene can reverse established glucocorticoid-induced pathology when administered after the onset of behavioral and immunological deficits, which would more closely model clinical scenarios where intervention begins after symptoms manifest.

## Conclusions

The present study demonstrates that lycopene effectively attenuates dexamethasone-induced depressive-like behaviors, neurochemical disturbances, oxidative-inflammatory stress, and systemic immunosuppression in rats. The protective effects are region-specific, with both hippocampus and Prefrontal cortex showing significant improvement, and encompass behavioral,, and cellular endpoints across multiple interconnected physiological systems. These findings position lycopene as a promising nutraceutical intervention for glucocorticoid-associated neuropsychiatric and immunological disturbances and support further investigation of its therapeutic potential in clinical populations requiring chronic glucocorticoid therapy. The multimodal mechanism of action simultaneously targeting oxidative stress, neuroinflammation, neurotrophic support through BDNF preservation, and immune function through lymphocyte protection may offer advantages over conventional agents with more limited mechanisms. Future research should focus on clinical translation through randomized controlled trials examining whether lycopene supplementation benefits patients with glucocorticoid-induced mood disturbances or stress-related depressive disorders, and whether these effects are mediated through the same molecular pathways identified in preclinical models.

## Data Availability

The authors confirm that the data (tables and figures) supporting the findings of this study are available within the article.

## Abbreviations

AChE: Acetylcholinesterase
ALT: Alanine Aminotransferase
ANOVA: Analysis of Variance
AST: Aspartate Aminotransferase
BDNF: Brain-Derived Neurotrophic Factor
CAT: Catalase
CD4: Cluster of Differentiation 4
CD8: Cluster of Differentiation 8
CNS: Central Nervous System
DEX: Dexamethasone
EDTA: Ethylenediaminetetraacetic Acid
ELISA: Enzyme-Linked Immunosorbent Assay
FST: Forced Swim Test
GABA: Gamma-Aminobutyric Acid
GSH: Reduced Glutathione
HOMA-IR: Homeostasis Model Assessment of Insulin Resistance
HPA: Hypothalamic-Pituitary-Adrenal
IFN-γ: Interferon-Gamma
IL-4: Interleukin-4
IL-6: Interleukin-6
IP: Intraperitoneal
MDA: Malondialdehyde
NF-κB: Nuclear Factor Kappa-B
NHANES: National Health and Nutrition Examination Survey
NO: Nitric Oxide
Nrf2: Nuclear Factor Erythroid-2-Related Factor 2
PBS: Phosphate-Buffered Saline
ROS: Reactive Oxygen Species
RONS: Reactive Oxygen and Nitrogen Species
SD: Standard Deviation
SEM: Standard Error of the Mean
SOD: Superoxide Dismutase
Th1: T-Helper Type 1
Th2: T-Helper Type 2
TNF-α: Tumor Necrosis Factor-Alpha
TST: Tail Suspension Test
WBC: White Blood Cell
WHO: World Health Organization
